# The Activity of Plant Crude Extracts against *Schistosoma mansoni*

**DOI:** 10.1155/2021/4397053

**Published:** 2021-11-09

**Authors:** Hirut Basha, Hassen Mamo

**Affiliations:** ^1^Ethiopian Public Health Institute, Addis Ababa, Ethiopia; ^2^Department of Microbial, Cellular and Molecular Biology, College of Natural and Computational Sciences, Addis Ababa University, PO Box 1176, Addis Ababa, Ethiopia

## Abstract

**Background:**

*Schistosoma mansoni* remains a significant health problem in low-income countries. Praziquantel (PZQ) is the only drug available to treat schistosomiasis, and PZQ resistance is a potential threat towards control of the disease although PZQ is currently effective against all species of schistosomes. Moreover, PZQ is less efficacious against larval stages. In response to these challenges, multiple *in vivo*/*in vitro* studies evaluated the anti*-S. mansoni* activity of crude plant extracts in a bid for novel drug(s). However, these studies appear fragmented and patchy. This systematic review explored the extent of such studies in the past 11 years (2010-2020).

**Methods:**

A systematic web search analysis and review of the literature on crude plant extracts tested against *S. mansoni* was done. Data from 17 articles meeting eligibility criteria were extracted and analyzed. Forty-three plant species have been tested by the 17 studies. The leaves, barks, stems, flowers, rhizomes, and roots of the plants as well as the whole plant part were used for the experiments.

**Conclusion:**

Nearly all of the plants significantly reduced schistosome egg output, killed adult worms, and improved liver histology and function. Further studies are required to assess the therapeutic potential of more promising plant species.

## 1. Introduction

Schistosomiasis is a top debilitating metazoan disease. It is caused by several species of the genus *Schistosoma* (phylum: Platyhelminths). Unlike the other trematodes that are liver, lung, or intestinal flukes causing an array of foodborne trematodiases [1], schistosomes are blood flukes and schistosomiasis (bilharzia or 'snail fever') is commonly referred to as a waterborne disease. Schistosomes, that are known since 1856 [2], have some additional peculiar biology within the class trematoda [3]. The three most important and most extensively investigated human *Schistosoma* species are *Schistosoma mansoni*, *S. haematobium*, and *S. japonicum* [4–6].

Although human schistosomiasis is mainly a chronic disease of the urinary bladder (caused by *S. haematobium*) and the small intestine (*S. japonicum*) or large intestine (*S. mansoni*), it has an acute phase as well. The most common symptoms of acute intestinal schistosomiasis are abdominal pain, diarrhea, and anemia [7]. The main symptoms of chronic intestinal schistosomiasis are linked to complex host immunological reactions to the parasites' egg antigens in the liver leading to a possible failure of this critical organ [8–10].

Remarkable success has been achieved against this longstanding disease mainly through targeted mass chemotherapy [11] following the prescription of single-dose praziquantel (PZQ)—sold under the brand name Biltricide among others—as first-line treatment since 2005. PZQ was discovered in the 1970s and was approved for human use in the United States of America in 1982 [12]. Nonetheless, schistosomiasis remains among the major global health threats. The World Health Organization (WHO) estimated that out of at least 290.8 million infected people in at least 78 countries, only 98.7 million were treated for schistosomiasis in 2017 [13]. Schistosomiasis is among the neglected tropical diseases (NTDs), and that is the main control challenge [14]. *S. mansoni* is widely distributed across the tropics/subtropics especially in the vast poverty-stricken but environmentally/climatically friendly sub-Saharan Africa [15–17].

The inadequate coverage of the desperately needed schistosomiasis preventive chemotherapy in Africa is largely attributable to cost [18]. On top of this, PZQ has other limitations. It has a reduced prophylactic effect at the recommended doses against immature stages [19, 20]. Furthermore, there is little data on PZQ safety and efficacy in preschool children leading to the exclusion of this age group from chemotherapy preventive control programs [21], and there is no oral formulation for infants and preschool children [22]. The drug has no effect if the liver and spleen are seriously affected.

Besides, although this drug appears safe and effective against all adult *Schistosoma sp* in general [23], an in-depth understanding of the efficacy and safety of various doses for different *Schistosoma* sp is necessary to extensively use PZQ. Some studies reported PZQ therapeutic failure [24], some up to 40% [25]. Above all, heavy dependence on the single available drug, which has been in use for over 40 years, is a source of serious concern. Drug resistance is an eventual scenario for any drug, and PZQ cannot be an exception forever [26]. Thus, there is a need to search for novel drugs against all lifecycle stages of the parasite with considerations for pediatric use. To eliminate schistosomiasis by 2030, the WHO calls for the development of new intervention tools including alternative drugs to PZQ [27].

Most modern drugs have their basis in traditional medicine. Nearly 30% or more of the modern pharmacological drugs are derived directly or indirectly from plants [28, 29]. People in low-income countries rely on traditional medicine for the treatment of schistosomiasis and other parasitic diseases. For example, in Ethiopia, a large number of communities, particularly in rural areas, rely on traditional medicinal plants to fight several diseases including schistosomiasis [30, 31]. Some of the reasons why people practice traditional medicine are the high cost of modern drugs, paucity and inaccessibility of modern health services, and cultural acceptability of traditional medicine [30]. In general, developing new drugs is an expensive and long process; therefore, focusing on medicinal plants that have already shown therapeutic efficiency could be the best strategy.

In light of this, studies assessed the activity of various medicinal plant crude extracts against *S. mansoni* with the hope of guiding novel antischistosomal drug development. However, there is scattered and erratic information on the diversity and identity of the antischistosome traditional medicinal plants and the efficacy of their different crude extracts against the various lifecycle stages of the parasite and the histopathology of schistosomiasis. This is, therefore, an attempt to produce a systematic review of the activities of different medicinal plants against the parasite.

## 2. Methods

### 2.1. Review Question and Literature Search

What is the extent of studies on medicinal plant crude extracts against *S. mansoni* and the efficacy of plant extracts against the parasite? A comprehensive electronic literature database search was conducted using four search engines (*Web of Science*, *Scopus*, *Embase*, and *PubMed*). The key terms used to search the literature were “schistosomiasis”, “schistosome”, “Schistosoma”, “bilharzia”, bilharziasis, “traditional medicine”, “medicinal plants”, and “crude plant extract”. These search key terms were connected with the Boolean operators (“AND”, “OR”) giving the algorithms “schistosomiasis” OR “schistosome” OR “Schistosoma” OR “bilharzia” OR bilharziasis AND “traditional medicine” OR “medicinal plant” OR “crude plant extract”. The literature review was done following the PRISMA (Preferred Reporting Items for Systematic Review and Meta-Analyses) guidelines [32].

### 2.2. Selection Criteria

Articles obtained from the initial search were screened based on defined inclusion and exclusion criteria. The inclusion criteria were that the study must be *in vivo* and/or *in vitro* on *anti-S. mansoni* activity of crude plant extracts, English language, and publication after 2009. The excluded studies were those that focused on *S. mansoni* cercariae only; that reported on plant fractions or compounds, and articles with no open access full text.

### 2.3. Screening Process and Data Extraction

Screening of search outputs was performed in three stages. In the first stage, titles were read to check the eligibility of the articles. At the second stage, abstracts were assessed for articles that fulfilled the first screening criteria. Finally, for articles that fulfilled the second screening criteria, full texts were downloaded and read for eligibility.

Data from the eligible articles were extracted using a predesigned format. The abstracted information included first author, title, publication year, area (country) where the study was conducted or the plant extracts originated, and type of research performed (*in vitro* or *in vivo*). The outcome parameters were egg reduction, juvenile and adult worm status including tegumental morphology, granuloma number and diameter, and liver function test. Other data extracted were information on phytochemical analysis, toxicity profile, plant species and tissue type used, and extraction solvent(s).

### 2.4. Quality Assessment

The qualities of the articles were assessed using SYRCLE's RoB tool [33]. Scores were given to each question, score “1” to “yes” and “0” to “no.” The highest score was 7 and the lowest 0. Articles with a total score of 6-7 were considered “high quality,” 3-5 “medium,” and 0-2 “low-quality” articles. Articles with “medium” and “high” quality were considered for analysis. Fortunately, none of the studies had “low-quality” score (0-2), so no study was excluded based on the quality assessment criterion.

## 3. Results

**Figure 1:**
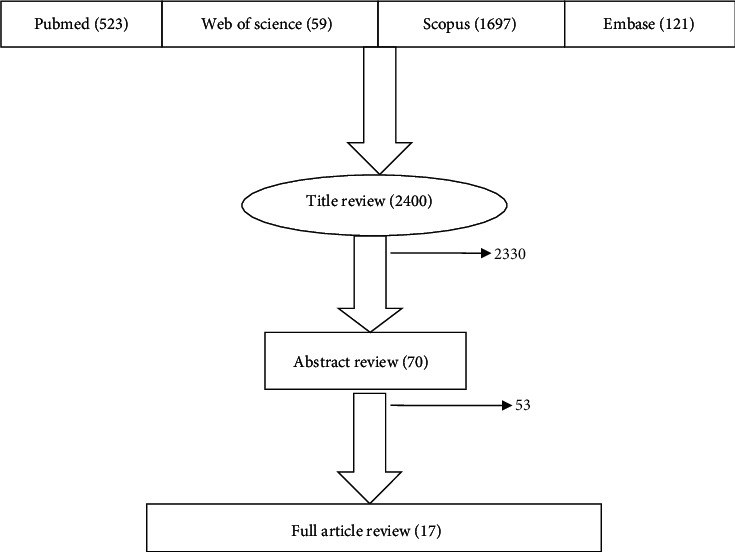
Article screening stages: PRISMA (Preferred Reporting Items for Systematic Review and Meta-Analyses) flowchart diagram of the record selection process.

From the search strategy, 2400 peer-reviewed articles were identified. Seventy articles were found relevant at first-level screening. Finally, 17 papers that were eligible for the set criteria were selected (Figure 1). Of these eligible studies, 6 were from Brazil, 5 from Egypt, 3 from Ghana, and 1 each from Cameroon, Kenya, and Ethiopia. In 2011 there were two studies, in 2014 four, in 2015 two, in 2016 one, in 2017 three, in 2018 one, in 2019 one, and in 2020 three. Five (29.4%) studies were *in vitro* plus *in vivo*, 8 (47.1%) *in vivo*, and 4 (23.5%) *in vitro*. Overall, the experiments involved 43 plant species with 10 studies on a single species, 4 on two, 2 on three, 1 on five, and 1 on twenty-four (Table 1). Six plant species were worked on by more than one study. *Azadirachta indica* was tested by four studies, two *in vitro* [34, 35], one *in vivo* [36], and the third *in vitro* plus *in vivo* [37]. *Phyllanthus amarus* [36, 38], *Vernonia amygdalina* [35, 36], *Rauwolfia vomitoria* [35, 39], and *Morinda lucida* and *Nauclea latifolia* [35, 36] were tested by two studies.

The other plants were *Mitracarpus frigidus* [40], *Ozoroa pulcherrima* [41], *Calotropis procera* [42], *Ficus elastica* [42], *Zingiber officinale* [42], *Punica granatum* [43], *Tanacetum vulgare* [44], *Allium sativum* (garlic) [45], *Allium cepa* (onion) [45], *Echinops kebericho* [46], *Hagenia abyssinica* [46], *Ageratum conyzoides*, *Alchornea cordifolia*, *Aloe vera*, *Alstonia boonei*, *Anthocleista nobilis*, *Combretum* sp, *Khaya senegalensis*, *Mangifera indica*, *Mitragyna stipulosa*, *Momordica charantia*, *Paulina pinnata*, *Phyllanthus niruri*, *Picralima nitida*, *Smeathmannia* sp, *Syzygium aromaticum*, *Tapinanthus bangwensis*, *Taraxacum officinale*, *Trichila monadelpha* and *Xylopia aethiopica* [35], *Arctium lappa* [47], *Baccharis trimera* [48], *Cynara scolymus* [49], *Ekebergia capensis* [37], *Chenopodium ambrosioides* [50], *Conyza dioscorides* [50], and *Sesbania sesban* [50].

Leaves, stem-barks, flowers, roots, rhizomes, or whole plant parts were used for the experiments. The most commonly used plant part was leaf (7/17 (41.2%)) followed by stem-bark (6/17 (35.3%), root (3/17(17.6%)) and flower (3/17 (17.6%)), aerial part (2/17 (11.7%)), pseudostem/rhizome (2/17 (11.7%)), and whole plant parts (1/17 (5.9%)). One study [50] did not indicate the plant tissue type used in its methods section. All of the plant extracts were prepared from dry plant materials. The extraction solvents were mostly methanol (41.2%) and ethanol (41.2%) followed by water (17.6%), dichloromethane (11.8%), ether (5.9%), acetone (5.9%), and hexane (5.9%), used in combination or solo (Table 2). Three studies [41–43] had done a preliminary phytochemical screening on five plant species, and the most frequently found phytochemical group was tannin followed by alkaloids and flavonoids (Table 3). Eight of the studies performed toxicity tests either *in vivo* or *in vitro* and declared the plants safe.

Almost all of the tested plants were reported to have significant antischistosomal activity. The plant extracts reduced juvenile and adult worm load and egg output. Moreover, worm motor activity, tegument morphology, coupling ability, and survival were significantly affected. Some of the extracts caused 100% worm mortality in a dose-dependent manner. The extracts also caused a significant reduction in granuloma number and size and and liver histopathology and improved liver function (Table 1).

Considering the findings, crude extracts of *A. sativum* and *A. cepa* normalized *S. mansoni*-associated increase in the levels of IgG, IgM, IL-2, IL-6, TNF-*α*, and catalase enzyme, accompanied by a decrease in GPX and SOD antioxidant enzyme activities in mice. Moreover, a significant reduction in worm burden, hepatic and intestinal eggs, and oogram count was noticed coupled with the restoration of mouse liver architecture [45].

*S. mansoni*-infected mice treated with methanol extract of *C. dioscorides*, *C. ambrosioides*, or *S. sesban* had worm load reduction rates of 40.9%, 53.7%, and 54.4%, respectively, 9 weeks postinfection. Adult worm and egg in the liver reduced to 66.3% and 76.9%, respectively, following successive treatment. Moreover, serum total protein and albumin levels and activities of alanine and aspartate transaminases and acid and alkaline phosphatases of infected treated mice improved in comparison with the negative control [50].

In a study from Brazil [48], crude dichloromethane extract and an aqueous fraction of *B. trimera in vitro* and *in vivo* similarly showed efficacy against schistosomula and juvenile and adult worms of *S. mansoni*. The exposure of the *in vitro* samples over adult parasites was able to inhibit 100% oviposition and mortality of the parasites with morphological alterations on the tegument and on the suckers, oral and acetabulum, in both males and females after 6-72 h of exposure. Overall, *B. trimera* exhibited major schistosomicidal effects *in vivo* against immature and adult worms of *S. mansoni*.

Fabri and colleagues [40] showed methanol extract of the aerial parts of *M. frigidus* opening the gynaecophoral canal of some male schistosomes and the presence of contorted muscles and vesicles and the darkening of the paired worm skin *in vitro*. The authors further noted that the extract significantly reduced total worm count, liver and spleen weight, and granuloma density *in vivo*. Similarly, the aerial parts of *T. vulgare* crude extract killed all adult worms, reduced viability and output of eggs, altered tegument morphology, and caused changes in the numbers of tubercles of *S. mansoni* male worms [44]. *A. indica* leaf ethanol extract caused a significant reduction in *S. mansoni* motor activity and severe tegument morphological and eventual death *in vitro*. The authors reported 100% adult worm mortality depending on dose and incubation time [34].

Leaf and stem-bark ethanol extracts of *P. granatum* separated coupled worms, reduced motor activity, altered tegument structure, and killed worms *in vitro. In vivo*, a significant reduction in hepatic granuloma number and diameter, eggs in liver tissues, liver inflammatory infiltration and hepatic fibrosis, and inducible nitric oxide synthase expression was noticed [43]. The stem-bark and roots of *R. vomitoria* were active against adult *S. mansoni* and were safe *in vitro* [39].

Ethanol extract of *A. lappa* fruit was safe to Vero cells and caused tegument morphological alterations including changes in the numbers of tubercles and significant reduction in motor activity and 100% mortality of adult *S. mansoni* [47]. The schistosomicidal activity of crude hexanoic and ethanolic extracts of *P. amarus* in *S. mansoni*-infected mice demonstrated against young adult and adult worms. The extract significantly reduced worm load as well as granuloma number and size [38].

*O. pulcherrima* root methanol extract against *S. mansoni* in mice caused significant reduction of worm burden and ova count in the feces, liver, and intestine. There was also a significant reduction of alanine aminotransferase activity as well as a significant increase of total protein content irrespective of dose. The total bilirubin level was also reduced and induced high malondialdehyde level irrespective of dose. Catalase activity and reduced glutathione concentration were significantly increased [41].

In a study that tested five Ghanaian medicinal plants [36], all of the plants exhibited varying adulticidal activities against *S. mansoni* in an exposure time- and concentration-dependent manner. The study evaluated the methanol crude extracts of the whole plant of *P. amarus*, leaves of *V. amygdalina* and *A. indica*, and both the leaves and bark of *M. lucida* and *N. latifolia in vitro* and *in vivo*. *A. indica-* and *V. amygdalina*-treated mice had significantly lesser mean liver and spleen weights compared to the untreated groups. *A. indica* demonstrated the highest adulticidal activities *in vitro*, whereas *V. amygdalina* exhibited the most potent adulticidal activity *in vivo*. Moreover, few to moderate granulomas were observed in the treatment groups compared to the untreated. Granulomas were significantly smaller in diameter in *V. amygdalina* and *A. indica* treatment groups than those in the untreated group. Moreover, treated infected mice had relatively less severe inflammatory cell infiltration.

Another group of Ghanaian investigators [35] considered 25 plant species that are used by traditional healers of schistosomiasis. The authors used these plants in formulations of 2-5 plant species in either aqueous herbal preparations or dried powdered forms and evaluated their *in vitro* activity against newly transformed schistosomula and adult worms of *S*. *mansoni*.

From East Africa, two studies were included in this systematic review as stated above. There were one each from Ethiopia and Kenya on different traditional medicinal plants. In Kenya, *S. mansoni*-infected mice treated with varying doses of aqueous extracts of *E. capensis* and *A. indica* showed a significant percentage reduction in worm burden and liver and intestine egg load in a dose-dependent fashion. Overall, *E. capensis* was more potent than *A. indica* [37]. In Ethiopia, crude methanol and ethanol extracts of *E. kebericho* root and *H. abyssinica* flower significantly reduced fecal egg count and adult worm burden in mice. The liver granuloma score of *E. kebericho*-treated infected mice was low, and the extract was safe for oral acute toxicity [46].

The only apparent negative reports or perhaps partial potency or toxicity of the plant extracts were those by two studies from Egypt. Leaf ethanol extract of *C. scolymus* did not exhibit significant effects on worm or tissue egg load and granuloma number *in vivo* although it caused a significant reduction of granuloma diameter and improvement of liver functions and liver fibrosis [49]. Furthermore, a study from Egypt [42] reported that *C. procera* latex and flower extracts were toxic, and *Z. officinale* extract did not significantly decrease worm load though after removal of the toxic rubber extract significantly reduced adult worm burden and tissue egg count and positively affected the oogram pattern.

## 4. Discussion

Many *in vivo*, *in vitro*, and *in vivo* and *in vitro* studies have been carried out to assess the efficacy of traditional medicinal plants that have widely been employed for schistosomiasis intervention in different endemic areas. However, these studies have not been critically appraised and their findings are not synthesized qualitatively or quantitatively. To our knowledge, the present study is the first systematic review of the antischistosomal activity of traditional medicinal plants.

In this review, the most commonly used plant part by traditional medicine practitioners was the leaf followed by the stem-bark and root. Leaf plays an important role in synthesizing secondary metabolites, which are a rich source of active chemical entities [51]. Moreover, one can easily harvest and extract leaves without causing harm to the surviving plants. This might be the reason why most traditional healers use leaves for herbal preparations. On the other hand, the constant use of plant stem-barks and roots for medicinal purposes poses risk to plant sustainability. Stem-bark or root harvesting needs greater care and replanting to assure sustainability of the plants.

From those studies that conducted phytochemical screening, tannins, flavonoids, and alkaloids were found in the majority of the plant extracts. Studies indicated that tannins, flavonoids, and alkaloids have antimicrobial [52] and antiparasitic [53] properties. Because of their antiparasitic property, these chemicals could be responsible for the schistosomicidal effect. It is worth noting that some of the medicinal plants were more effective than PZQ in killing the egg and adult worm. In addition to their antiparasitic properties, these compounds have antioxidants and anti-inflammatory properties [54]. Flavonoids and tannins can play an important role by releasing antioxidant effects against the damage of the liver membrane infected by the schistosomes.

Despite the promising antischistosomal performance of traditional medicinal plants, so far none could be developed as an alternative drug to treat schistosomiasis. There is a wide gap regarding research focused on identifying plant bioactivities and establishing the efficacy and safety of medical plants involving *in vivo* assay on higher animal models and randomized clinical trials for various reasons. To translate promising plants into safe, effective, and affordable alternative antischistosomal drugs, a collaboration between government bodies, researchers, traditional healers, and prospective business investors is needed.

## 5. Conclusion

Various authors from different countries recorded several plants having the potential for anti-*S. mansoni* drug development. However, nearly half of the studies lacked information on the potential side effects of the plants and further studies are required. Validation of the therapeutic potential as well as the safety profiles of more promising plant species like *A. indica*, which are tested by multiple authors, is necessary.

## Figures and Tables

**Table 1:** Data extracted from the selected articles.

Study	Country	Experiment	Plant species	Plants	Plant part	Outcomes
[50]	Egypt	*In vivo*	*C. dioscorides*, *C. ambrosioides*, *S. sesban*	3	?	Reduction in worm and egg loads, improved liver function
[45]	Egypt	*In vivo*	*A. cepa*, *A. sativum*	2	Pseudostem	Increased IgG, IgM, IL-2, IL-6, and TNF-*α* and lower catalase, GPX, and SOD antioxidant enzyme activities, worm burden, hepatic and intestinal eggs
[44]	Brazil	*In vitro*	*T. vulgare*	1	Leaf	Reduced egg output, motor activity, adult death
[42]^∗^^±^	Egypt	*In vivo*	*C. procera* ^a^, *F. elastic*, *Z. officinale*	3	Stem-latex, rhizome, flower	Reduced hepatic and intestinal tissue egg loads, adult worm burden, positively affected oogram pattern
[48]^ȱ±^	Brazil	*In vivo*\*vitro*	*B. trimera*	1	Leaf	Reduction in worm burden and eggs
[40]	Brazil	*In vivo*\*vitro*	*M. frigidus*	1	Aerial parts	Reduced worm count & contorted muscles, vesicles, and darkened paired worm skin
[34]	Brazil	*In vitro*	*A. indica*	1	Leaf	100% mortality of female worms, significant motor activity reduction, severe morphological changes
[37]	Kenya	*In vivo*	*E. capensis*, *A. indica*	2	Stem-bark, leaf	% worm load reduction, reduced tissues (liver and intestine) and egg load counts, the activity of the two plant extracts was dose-dependent with *E. capensis* being more potent in reducing both the worm burden at all the stages and tissue egg load
[43]^∗^^±^	Egypt	*In vivo\vitro*	*P. granatum*	1	Leaf, stem-bark	Adult death, reduced motor activity, reduced granulomas
[49]	Egypt	*In vivo*	*C. scolymus*	1	Leaf	Reduction of granuloma diameter, improvement of liver functions and liver fibrosis
[39]	Ghana	*In vivo\vitro*	*R. vomitoria*	1	Stem-bark, root,	Adult worm death
[47]^±^	Brazil	*In vitro*	*A. lappa*	1	Flower	No cytotoxic to Vero cells, 100% mortality and reduction on motor activity of all adult worms, tegumental morphological alterations and changes in the numbers of tubercles of adult worms
[38]	Brazil	*In vivo*	*P. amarus*	1	Leaf	Worm reduced, no alteration in the liver function
[46]	Ethiopia	*In vivo*	*E. kebericho*, *H. abyssinica*	2	Root, flower	Reduction of fecal egg count and adult worm burden, reduction in liver granuloma score
[41]^∗^	Cameroon	*In vivo*	*O. pulcherrima*	1	Root	Reduced ova, adult worm
[36]	Ghana	*In vivo*/*vitro*	*P. amarus*, *V. amygdalina*, *A. indica*, *M. lucida*, *N. latifolia*	5	Whole plant, leaf, stem-bark	Reduced mean liver and spleen weights, high adulticidal activities *in vitro*/*vivo*, fewer granulomas, smaller granuloma diameter
[35]	Ghana	*In vitro*	24 *A. indica* included	25^∗∗^	Almost all parts mentioned above	Inhibition of the motility of NTS^+^ and adult worm

^∗^Preliminary phytochemical screening done; ^±^acute toxicity done; ^ȱ^fresh specimen; ^a^was toxic before removing the rubber; ^+^newly transformed schistosomule; ^∗∗^a total of 25 plants in 7 groups in combinations of 2-5 plants in each group.

**Table 2:** Plant species, status of tissue used, extraction solvent, toxicity, and phytochemical tests.

Study	Plant species	Plant tissue status	Phytochemical screening	Toxicity test	Extraction solvent
[50]	*C. dioscorides*, *C. ambrosioides*, *S. sesban*	Dry	No	Yes	Methanol
[45]	*A. cepa*, *A. sativum*	Dry	No	No	?^∗∗^
[44]	*T. vulgare*	Dry	No	No	Ethanol, water
[42]^∗^^±^	*C. procera* ^a^, *F. elastic*, *Z. officinale*	Dry	Yes	Yes	Methanol, water, ether
[48]^ȱ±^	*B. trimera*	Dry	No	Yes	Dichloromethane, acetone
[40]	*M. frigidus*	Dry	Yes^∗^	Yes	Methanol
[34]	*A. indica*	Dry	No	No	Ethanol
[37]	*E. capensis*, *A. indica*	Dry	No	No	?^∗∗^
[43]^∗^^±^	*P. granatum*	Dry	No	Yes	Ethanol
[49]	*C. scolymus*	Dry	No	No	Methanol
[39]	*R. vomitoria*	Dry	No	Yes	Ethanol
[47]^±^	*A. lappa*	Dry	Yes^∗^	Yes	Ethanol, water
[38]	*P. amarus*	Dry	No	No	Hexane, ethanol
[46]	*E. kebericho*, *H. abyssinica*	Dry	No	Yes	Methanol, ethanol
[41]^∗^	*O. pulcherrima*	Dry	Yes	No	Methanol
[36]	*P. amarus*, *V. amygdalina*, *A. indica*, *M. lucida*, *N. latifolia*	Dry	No	No	Methanol
[35]	24 *A. indica* included	Dry	No	No	Dichloromethane, methanol

^∗^Compounds isolated; ^∗∗^extraction solvent not indicated in the methods section.

**Table 3:** Preliminary phytochemical screening profile of five plants studied by the articles assessed.

Plant species	Parts	Phytochemicals									
*O. pulcherrima*	Root	Tannins	Flavonoids	Alkaloids	Triterpenes	Saponins	Anthraquinones	Terpenoids	Phenols	Cardiac glycoside	Lipids
*P. granatum*	Stem, leaf	Tannins	Flavonoids	Alkaloids	Triterpenes	Polyphenol glycosides	Sterols	Anthocyanins	Triglycerides	NA	NA
*C. umbellatum*	Leaf	Tannins	Flavonoids	Alkaloids	Triterpenes	Saponins	Saponosides	NA	NA	NA	NA
*Z. officinale*	Root	Tannins	Flavonoids	Alkaloids	Triterpenes	Saponins	Sterols	NA	NA	NA	NA
*C. procera*	Flower	Tannins	Sterols	Terpenes	NA	NA	NA	NA	NA	NA	NA

Note: NA: no other chemical compounds were found.

## Data Availability

All data extracted or analyzed during this study are included in this manuscript.

## Abbreviations

SYRCLE's: Systematic Review Centre for Laboratory Animal Experimentation
IgG: Immunoglobulin G
IgM: Immunoglobulin M
IL-2: Interleukin 2
IL-6: Interleukin 6
TNF-*α*: Tumor necrosis factor alpha
GPX: Glutathione peroxidase
SOD: Superoxide dismutase
INOS: Inducible nitric oxide synthase
PZQ: Praziquantel
AST: Aspartate aminotransferase
ALT: Alanine aminotransferase.
